# Effectiveness of a digital medication event reminder and monitor device for patients with tuberculosis (SELFTB): a multicenter randomized controlled trial

**DOI:** 10.1186/s12916-022-02521-y

**Published:** 2022-09-28

**Authors:** Tsegahun Manyazewal, Yimtubezinash Woldeamanuel, David P. Holland, Abebaw Fekadu, Vincent C. Marconi

**Affiliations:** 1 Addis Ababa University, College of Health Sciences, Center for Innovative Drug Development and Therapeutic Trials for Africa (CDT-Africa), P.O. Box 9086, Addis Ababa, Ethiopia; 2 Emory University School of Medicine and Rollins School of Public Health, Atlanta, Georgia 30322 USA

**Keywords:** Tuberculosis, Medication event reminder monitor (MERM), Self-administered therapy, Directly observed therapy (DOT), Adherence, Treatment, Urine isoniazid testing, Digital health, Clinical trial, Ethiopia

## Abstract

**Background:**

Tuberculosis remains the leading cause of death from a single infectious disease worldwide. Trials evaluating digital adherence technologies for tuberculosis in low- and middle-income countries are urgently needed. We aimed to assess whether a digital medication event reminder and monitor (MERM) device-observed self-administered therapy improves adherence and treatment outcomes in patients with tuberculosis compared with the standard in-person directly observed therapy (DOT).

**Methods:**

We did a two-arm, attention-controlled, effectiveness-implementation type 2 hybrid, randomized controlled trial in ten healthcare facilities in Addis Ababa, Ethiopia. We included adults with new or previously treated, bacteriologically confirmed, drug-sensitive pulmonary tuberculosis who were eligible to start anti-tuberculosis therapy. Participants were randomly assigned (1:1) to receive a 15-day tuberculosis medication supply in the evriMED500® MERM device to self-administer and return every 15 days (intervention arm) or visit the healthcare facilities each day to swallow their daily dose with DOT by healthcare providers (control arm). Both arms were followed throughout the standard two-month intensive treatment phase (2RHZE). For control participants, some provider-approved take-home doses might be allowed for extenuating circumstances in real-world practice. Data were collected on patient information (demographic, socioeconomic, behavioral, social, and clinical information), medication adherence measures (MERM vs. DOT records, IsoScreen^TM^ urine colorimetric isoniazid test, and adherence self-report), and clinical measures (pre-post treatment sputum Xpert MTB/RIF assay or microscopy, and adverse treatment outcomes). The intention-to-treat (ITT) primary endpoints were (1) individual-level percentage adherence over the two-month intensive phase measured by adherence records compiled from MERM device vs. DOT records that also considered all take-home doses as having been ingested and (2) sputum smear conversion following the standard two-month intensive phase treatment. Secondary endpoints were (1) individual-level percentage adherence over the two-month intensive phase measured by adherence records compiled from the MERM device vs. DOT records that considered all take-home doses as not ingested, (2) negative IsoScreen urine isoniazid test, (3) adverse treatment outcome (having at least one of the three events: treatment not completed; death; or loss to follow-up), and (4) self-reported adherence. The MERM device has an electronic module and a medication container that records adherence, stores medication, emits audible and visual on-board alarms to remind patients to take their medications on time and refill, and enables providers to download the data and monitor adherence.

**Results:**

Participants were enrolled into the study between 02 June 2020 and 15 June 2021, with the last participant completing follow-up on 15 August 2021. A total of 337 patients were screened for eligibility, of whom 114 were randomly assigned and included in the final analysis [57 control and 57 intervention participants]. Participants were 64.9% male, 15% with HIV, 10.5% retreatment, and 5.3% homeless. Adherence to TB medication was comparable between the intervention arm [geometric mean percentage (GM%) 99.01%, geometric standard deviation (GSD) 1.02] and the control arm [GM% 98.97%, GSD 1.04] and was within the prespecified margin for non-inferiority [mean ratio (MR) 1.00 (95% CI 0.99–1.01); *p* = *0.954*]. The intervention arm was significantly superior to the control arm in the secondary analysis that considered all take-home doses in the control were not ingested [control GM% 77.71 (GSD 1.57), MR 1.27 (95% CI 1.33–1.43)]. Urine isoniazid testing was done on 443 (97%) samples from 114 participants; 13 participants had at least one negative result; a negative test was significantly more common among the control group compared with the intervention group [11/57 (19.3%) vs 2/57 (3.5%); *p* = *0*.*008*]. There was no significant difference between the control and intervention arms for smear conversion [55 (98.2%) vs 52 (100%); *p>0.999*], adverse treatment outcomes [0 vs 1 (1.9%); *p* = *0.48*], and self-report non-adherence [5 (8.9%) vs 1 (1.9%); *p* = *0*.*21*].

**Conclusions:**

In this randomized trial of patients with drug-sensitive pulmonary tuberculosis, medication adherence among participants assigned to MERM-observed self-administered therapy was non-inferior and superior by some measures when compared with the standard in-person DOT. Further research is needed to understand whether adherence in the intervention is primarily driven by allowing self-administered therapy which reduced challenges of repeated clinic visits or by the adherence support provided by the MERM system. To avoid contributing to patient barriers with DOT, tuberculosis medical programs should consider alternatives such as medication event monitors.

**Trial registration:**

ClinicalTrials.gov, NCT04216420.

**Supplementary Information:**

The online version contains supplementary material available at 10.1186/s12916-022-02521-y.

## Background

Tuberculosis (TB) continues to be a leading cause of death from a single infectious disease worldwide, with an estimated 10 million people falling ill and 1.5 million people dying from TB each year [1]. The long course and complexity of anti-TB therapy result in poor adherence to medications, poor treatment outcomes, and drug resistance [2–5]. In-person directly observed therapy (DOT) has long been in use to assure adherence through patients swallowing daily doses under direct observation at a healthcare facility; however, there have been key ethical, social, and economic issues that disrupt the DOT process [5–8]. Debates continue about what particular technologies could improve adherence to TB medication and treatment outcomes that do not require in-person DOT.

Digital adherence technologies (DATs) are emerging as promising patient-centered solutions to avert the problems with in-person DOT by remotely monitoring and reminding patients to take their TB medications in a convenient location. Electronic medication monitors are attracting attention in this regard and efforts are underway to establish robust efficacy and effectiveness profiles across different population groups. A pragmatic stepped-wedge cluster-randomized trial reported a similar odds of TB treatment success with a 99DOTS-based electronic medication monitor compared with DOT largely provided by family treatment supporters [9]. A pragmatic cluster-randomized trial reported improved medication adherence with the use of medication event reminder and monitor compared with DOT, but not with text messaging [10]. Another randomized controlled trial reported a non-inferior adherence to TB medication with the use of wirelessly observed therapy with an edible ingestion sensor compared with DOT [11]. Trials supporting electronic medication reminders are, in general, limited and show conflicting results with heterogeneous outcomes, providing a basis for the relevance of the current study. Some observational studies also assessed the potential use of electronic medication monitors for patients with TB but with inconsistent selection of outcome variables and contradictory findings. The findings demonstrated suboptimal accuracy [12] no improvement in TB treatment outcomes [13], satisfactory uptake [14], implementation challenges but reduced stigma [15], improved adherence [16], a higher treatment success rate [17], and higher user acceptability [18–20]. The World Health Organization (WHO) recently recommended that countries maximize the use of DATs to complement DOT as programmatic uptake of such technologies remains suboptimal [21].

Ethiopia, the second-most populous country in Africa with a population of 120 million (2022), is among the 30 high-burden countries for TB and HIV-associated TB according to the WHO Global TB report 2021 [1]. TB is still the third leading cause of death among communicable, maternal, and neonatal diseases in the country. Several studies carried out in Ethiopia reported that patients with TB and their providers see the standard in-person DOT as a very burdensome strategy [22–25]. As a result, DOT survives in principle, while implementation is irregular as both patients and providers have ambiguities about the program. Its rigid implementation could even lead to the emergence of more drug-resistant TB - a challenge DOT was invented to resolve [26].

In this trial, we aimed to test the hypothesis that the use of a digital medication event reminder and monitor device-observed self-administered therapy provides a non-inferior medication adherence and treatment outcomes for patients with TB compared with the standard in-person DOT in Ethiopia, one of the low-income countries with the highest burden of TB.

## Methods

### Study design and participants

This is a multicenter, two-arm randomized, attention-controlled, non-inferiority, effectiveness-implementation type 2 hybrid trial done in 10 healthcare facilities in Ethiopia. The trial is registered with ClinicalTrials.gov, NCT04216420, and a full description of the study protocol [5] (Additional file 1) and a systematic review in support of the trial [27] were published elsewhere. As part of the trial, cross-sectional mixed-methods studies have been published elsewhere that assessed the capacity and readiness of the study sites to adopt and implement new digital health interventions for TB [28] and that assessed providers’ perceptions and acceptability of digital health interventions in the clinical care and treatment of TB [29]. The trial CONSORT checklist is available in Additional file 2.

Eligible potential patients were adults aged ≥ 18 years; new or previously treated, bacteriologically confirmed drug-sensitive pulmonary TB; eligible to start the standard 6-month first-line anti-TB medication; from the outpatient setting; and willing and able to provide informed consent. Patients were ineligible if they had known drug resistant-TB or if they had a concurrent health condition that precluded informed consent or safely participating in the study procedures.

**Fig. 1:**
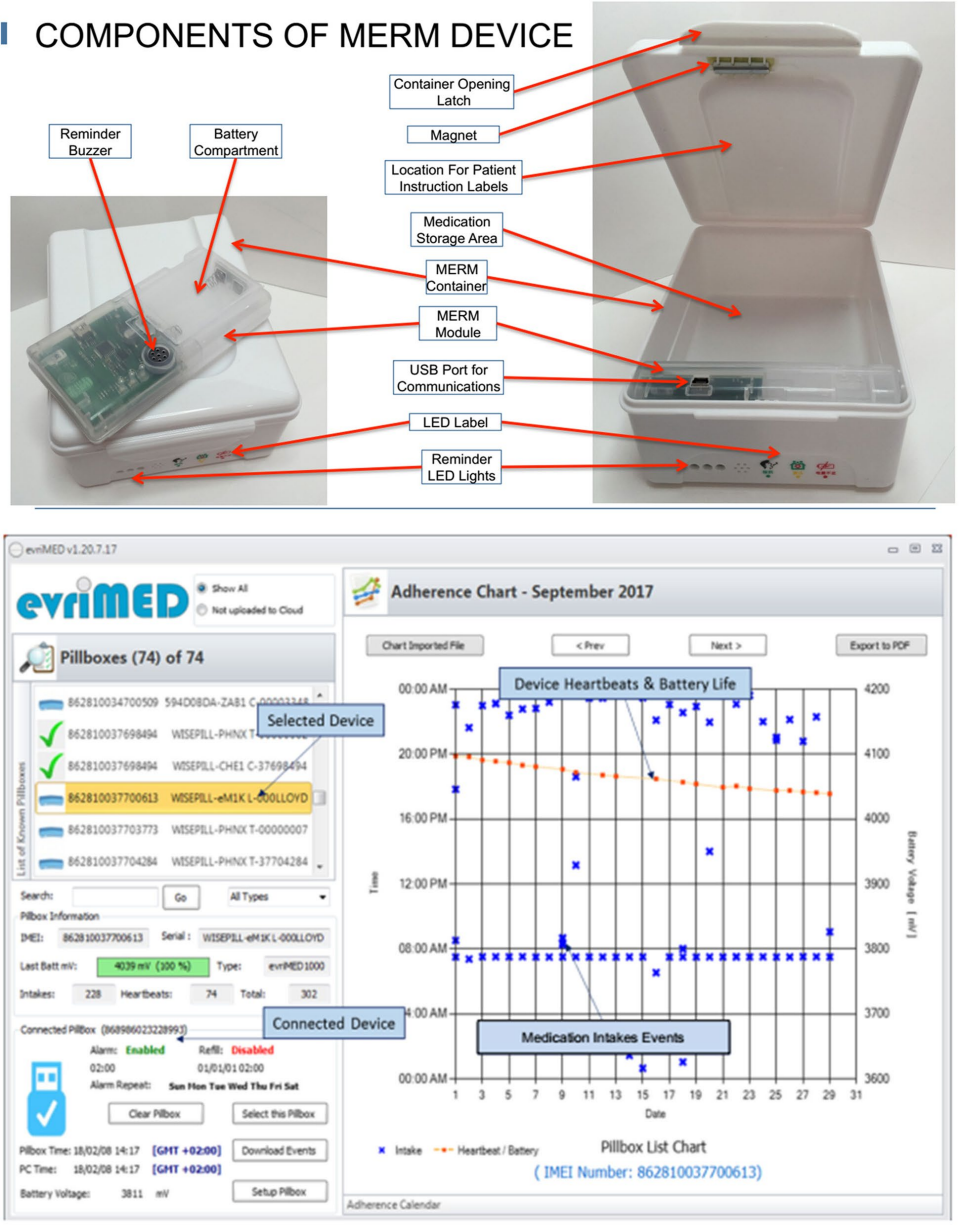
Components and application of the evriMED500 medication event reminder and monitor system [18, 30]

The trial of interest in this study was the Medication Event Reminder Monitor System (MERM), evriMED500®, manufactured by Wisepill Technologies, South Africa [30]. The evriMED500 dispenser consists of a medication container and an electronic module with slots in the container so that the indicator light-emitting diodes (LEDs) are visible through the front of the container (Fig. 1). It is an electronic pillbox that records adherence to treatment, stores medication, emits audible and visual alerts to remind patients to take their medications, and enables healthcare providers to monitor adherence. There are three indicator LEDs. For the daily medication reminder, a green LED flashes once when the container is opened and again once when the container is closed; quickly flashes three times when the container is opened and closed quickly; flashes in sequence during the daily medication alarm; and remains solid while connected via USB to the computer. For the medication refill reminder, a yellow LED flashes along with the green LED at the time of the medication alarm. If the medication alarm is not enabled, only the yellow LED flashes and is on solid when the container is opened. For low-battery alerts, a red LED flashes along with the green LED at the time of the medication alarm and remains solid when the container is opened. The dispenser has a buzzer that is activated during the alarm sequences, and it emits a soft tone when the container is opened or closed. It also holds a USB data port for downloading data and for the configuration of the unit. MERM supports treatment for TB as well as other co-infections. It is currently being tested in trials and is being used in some countries [30]. The device is described further in the study protocol [5]. The trial’s primary investigator and the trial coordinators have received training on the application and use of the Wisepill evriMED® technology by the developer company, Wisepill Technologies, South Africa. Importation of all study-related devices, reagents, and supplies was reviewed and certified by the authority responsible by law (the Ethiopian Food and Drug Authority).

### Randomization and masking

Consented participants were randomly assigned (1:1) to either MERM (intervention arm) or DOT standard care (control arm) using a computer-generated random number sequence developed by a trial expert who did not participate in recruitment. Permuted block randomization method was used to randomly allocate participants and maintain a balance of the number of participants assigned to each arm. The study investigators who were responsible for assessing study outcomes and writing the report were blinded to group allocation until the manuscript was completed. Because of the scope of the trial, participants and the other study staff were not blinded to group allocation. A statistician masked to group allocation performed the analyses. No stratification was needed for key variables.

### Procedures

The main data collection tools included a baseline patient information questionnaire (demographic, socioeconomic, behavioral, social, and clinical information), medication adherence measurement tools (MERM vs. DOT daily treatment adherence monitoring tool and urine colorimetric isoniazid test - IsoScreen^TM^ test, GFC Diagnostics Ltd, Bicester, England [31], and adherence self-report questionnaire) and clinical measurement tools (pre-post treatment sputum Xpert MTB/RIF assay or microscopy and adverse treatment outcome monitoring tool). A standard operating procedure was developed for each activity and placed at each study site following the review and approval by the implementing healthcare providers.

The healthcare provider clearly described to the study participant the information provided in the study information sheet and the consent form in a way that was understandable, providing ample time and ensuring that the informed consent was well understood. If the participant agreed to participate, the healthcare provider contacted the study trial coordinator for the result of the randomization, whether the participant was assigned the MERM-observed self-administered therapy or DOT arm, and informed the participant of the assignment. The provider gave a copy of the information sheet and a signed copy of the consent form to the study participant. Then, the provider documented all required information and data from the consent form to the study logbook: date, serial number, full name, participant identification number, study arm, and identification number of the healthcare provider. The provider retained the original completed consent form and the study logbook separately from participant medical charts in a locker cabinet to ensure that only the provider and the study staff could have access. For each participant in both arms, the provider collected baseline information, including demographic, socioeconomic, behavioral, and social characteristics using a semi-structured questionnaire (Additional file 3).

Both arms were followed throughout the intensive treatment phase that lasts two months for drug-susceptible TB. Treatment was based on the WHO-recommended two-month fixed-dose-combination of first-line anti-TB drug delivered as a single daily dose, 2RHZE (rifampicin [R]/150 mg + isoniazid [H]/75 mg + pyrazinamide [Z/400 mg + ethambutol ([E]/275 mg). Treatment follow-up was made by the full-time healthcare providers in the TB clinic following a moderate on-site orientation and the Wisepill evriMED application was set up on computers that have already been in use in TB or similar clinics to understand the sustainability of the intervention in a broad sense.

Participants in the intervention arm were informed on how to use the evriMED500 device and given a graphical leaflet prepared in the local national language outlining the procedures (Additional file 4). The orientation time depended on the efficiency of the participants to fully acquire and demonstrate the necessary skills. Based on the Wispill evriMED® user manual, the provider opened the container, removed the MERM Module from the designated area in the MERM container, inserted batteries to activate the MERM module, and connected it to the computer via the USB cable. The provider asked the participant for a convenient time to ingest the medicine, preferably in the morning adhering to the national guidelines, and configured the module with the date and time, the medication reminder time, and the medication refill reminder alert using the pillbox application. Once the MERM Module setup was completed, the provider disconnected it from the computer and placed it back in the designated slot of the MERM container. Then, the provider added the patient instruction label inside the MERM device, placed a 15-day TB medication supply (HRZE fixed-dose combination therapy of 15 doses) in the medication storage area of the MERM device, and closed the container. The entire device was then given to the participant for self-administration of the medications. The participants returned every 15 days, where the healthcare provider counted any remaining tablets in the pillbox and connected the MERM module with the computer. Along with the participant, the provider downloaded the pill-taking data from the Wisepill® device to the computer and reviewed the event reports over the previous 15 days. This included the dates and times that the user opened the device, to define how adherent the user was to the prescribed ingestion times. Any missed event, where no ingestion occurred over a particular prescribed ingestion period in the event report, was evaluated against any remaining tablets in the pillbox and discussed further with the participant for confirmation. With these, using the study’s paper-based daily treatment adherence monitoring tool (Additional file 5), the provider completed the information on daily medication adherence and reasons for any missed doses. All treatment guidance, counseling, and promotion measures were provided in a similar fashion as the standard DOT control procedures. Any participant in the intervention arm who delayed (>15 days) for a follow-up was considered non-adherent for each day the patient did not refill medications. Any participant in the intervention arm who missed more than five tablets in any 15-day refill period was subject to reassignment to DOT throughout the remaining days of the intensive phase. The provider also evaluated the functionality of the device with troubleshooting as needed.

The participants underwent IsoScreen^TM^ urine isoniazid test, which is a colorimetric assay, whereby the pyridine ring structure of isoniazid and its metabolites is broken by the biochemical reaction leaving it vulnerable to attachment by the condensing agent, barbituric acid. This forms a colored derivative. Results were interpreted as positive (treatment adherence) or negative (treatment non-adherence) based on observed color following the mixture of the urine specimen with the dried reagent in the reaction chamber. A dark blue or black color (drug was taken within the last 24–30 h) or green color (drug was taken but within the last 30–48 h) were interpreted as isoniazid positive and urine color not changed (drug not taken or potentially taken longer than 48 h) was interpreted as isoniazid negative. The kit was procured directly from the manufacturer, GFC Diagnostics Ltd, Bicester, England, for assurance of proper shipping and logistics.

Participants in the control arm were managed according to the standard DOT practice, where they visited the healthcare facility each day throughout the 2-month intensive phase to swallow their daily dose of RHZE with direct observation by TB healthcare providers. The healthcare providers filled out a similar adherence monitoring tool where medication adherence could be calculated accordingly. Urine samples for isoniazid testing were collected and performed every 15 days as also done for the intervention arm.

For the control arm, in addition to monitoring medication adherence, we conducted a separate secondary analysis for any doses that were self-administered after approval from their provider to determine if this approach had an impact on other outcome measures. This was to determine the real-world practice of in-person DOT where some doses might be self-administered when the provider approved this procedure for extenuating circumstances.

At the end of the intensive phase, participants underwent microbiological testing to assess sputum smear conversion. A trained study staff completed several data tools for both arms, including a treatment outcome monitoring tool, adherence self-report, a side effect reporting, and clinical laboratory test results in addition to TB diagnostics. For self-reported medication adherence, participants were asked questions about the relationship between treatment adherence and stress over meeting their TB treatment schedule. These were driven by two key questions: “Over the past 2 months, did you often forget to take your TB medications?” and “Did you feel stressed about meeting the TB medication schedule?”

### Primary outcomes

The intention-to-treat (ITT) primary endpoints were (1) individual-level percentage adherence averaged over the two-month intensive phase measured by adherence records compiled from the MERM device vs. DOT records assuming all take-home doses were ingested, following pill counts for any missed pills in both arms, and (2) sputum smear conversion following the standard 2-month intensive phase treatment based using a non-inferiority design.

### Secondary outcomes

The secondary endpoints were (1) individual-level percentage adherence averaged over the 2-month intensive phase measured by adherence records compiled from the MERM device vs. DOT records assuming all take-home doses were not ingested, (2) negative urine isoniazid test, defined as participants having at least one negative test result, (3) adverse treatment outcomes, defined as participants having at least one of the three events: treatment not completed; death; or loss to follow-up, and (4) self-reported adherence, defined as participants who forgot to take their medication. The implementation outcomes stated in the protocol [5] including health-related quality of life, cost, treatment satisfaction, and usability of the intervention will be reported separately.

### Statistical analysis

The sample size was calculated considering a 1-sided type I error of 2.5%, a power of 80%, 10% attrition rate, delta (non-inferiority margin) of 20%, and a continuous outcome of percentage adherence over the 2-month intensive phase, with a standard deviation of 36% and 79% of average adherence [32], assuming null hypothesis for both arms. The results yield a sample size of 57 in each arm for a total of 114 participants. For the primary outcome, we did a log_10_ transformation on the data, where a difference can be equivalently transformed into a ratio using a power (10^a^). The non-inferiority was calculated as the log in the control minus the log in the intervention, which was equivalent to the log of the control divided by intervention. Descriptive summary measures were used to report participant characteristics. Chi-square tests were used to evaluate potential associations among categorical variables. To compare the level of adherence between study arms and among variables, independent *t*-tests were done on log-transformed adherence percentage of the expected 60 days. Effects of the arms and other adherence variables were estimated using a geometric mean (GM) with geometric standard deviation (GSD) and mean ratios (MR) with 95% confidence intervals (CI). Log binomial regression was conducted to see the effect of study arms on at least one negative isoniazid urine test and self-reported adherence of participants. Effects were measured using relative risk (RR) with 95%CI. A general linear model was done on log-transformed adherence percentage to identify the effects of variables on participants’ level of adherence. Effects were measured using an adjusted mean ratio (AMR) with 95% CI. A sensitivity analysis was done for the adherence measurement considering pill count as the outcome; thus, the number of pills taken during the 2 months intensive period. Poisson regression and negative binomial regression were done to identify factors that determine participants’ level of adherence. In all analyses, a 5% significance threshold was used to determine statistical significance.

## Results

### Baseline characteristics

**Fig. 2:**
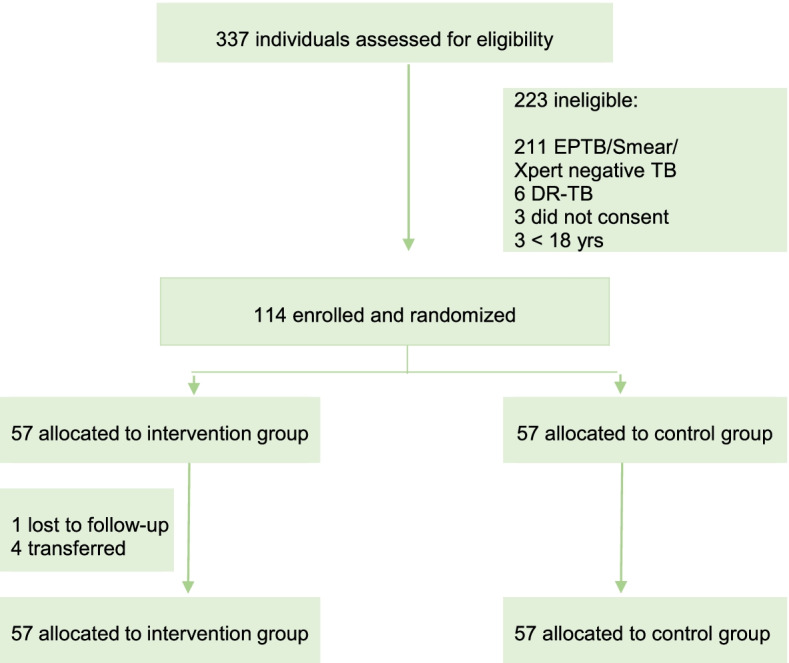
SELFTB CONSORT trial diagram

Participants were enrolled into the study between 02 June 2020 and 15 June 2021, with the last participant completing follow-up on 15 August 2021. A total of 337 patients from 10 healthcare facilities in Addis Ababa, Ethiopia, were screened for eligibility and, of these, 114 were selected, randomly assigned 1:1 into the trial with 57 (50%) to intervention and 57 (50%) to control arms, and included in the final analysis. The most frequent reasons for exclusion were status as extrapulmonary TB, smear/Xpert-negative, and drug-resistant TB on the initial TB diagnostic test (Fig. 2). From the intervention arm, four participants were transferred and one was lost to follow-up; the analysis assumed complete non-adherence throughout the remaining time where the participants missed their refills.

The mean age of the participants was 32.9 years (standard deviation [SD] 11.07) and 74 (64.9%) were male. Twelve (10.5%) were retreatment cases and had completed their previous treatment, and 17 (15%) had HIV infection, of whom 12 (70.6%) were on antiretrovirals. Laboratory diagnostic tools for pre-treatment confirmation of TB were the Xpert MTB/RIF assay [70 (61.4%) participants] and acid-fast bacillus smear microscopy [44 (38.6%) participants]. Among the participants diagnosed with pulmonary TB using smear microscopy, 16 (38.1%) were graded 3+ and 15 (35.7%) 2+. The mean monthly income was Ethiopian Birr (ETB) 2959.21 (SD ETB 3160, min. ETB 0 and max. ETB 15000), 6 (5.3%) were homeless, 72 (63.2%) lived in a house with a single bedroom, and 20 (17.5%) smoked cigarettes. Baseline characteristics and HIV status were balanced between the two groups (Table 1). During the intensive phase, participants were prescribed a standard first-line ant-TB regimen, and none of the participants in either group permanently discontinued treatment. Drug supply issues were not reported.

**Table 1:** Characteristics of study participants (*n* = 114)

Variables	Categories	***f*** (%)	Arm	
			Intervention ***f*** (%)	Control ***f*** (%)
**Gender**	Female	40 (35.1)	18 (31.6)	22 (38.6)
	Male	74 (64.9)	39 (68.4)	35 (61.4)
**Marital status**	Never	54 (47.4)	27 (47.4)	27 (47.4)
	Married	51 (44.7)	29 (50.9)	22 (38.6)
	Widowed	2 (1.8)	0 (0)	2 (3.5)
	Divorced	7 (6.1)	1 (1.8)	6 (10.5)
**Occupation status**	No job	23 (20.2)	10 (17.5)	13 (22.8)
	Student	4 (3.5)	4 (7.0)	0 (0)
	Farmer	1 (0.9)	0 (0)	1 (1.8)
	Trader	10 (8.8)	5 (8.8)	5 (8.8)
	Housewife	9 (7.9)	2 (3.5)	7 (12.3)
	Government employee	10 (8.8)	5 (8.8)	5 (8.8)
	Daily laborer	45 (39.5)	22 (38.6)	23 (40.4)
	Other	12 (10.5)	9 (15.8)	3 (5.3)
**Highest level of education**	No formal education	9 (7.9)	3 (5.3)	6 (10.5)
	Primary	44 (38.6)	25 (43.9)	19 (33.3)
	Secondary	30 (26.3)	14 (24.6)	16 (28.1)
	Preparatory	11 (9.6)	5 (8.8)	6 (10.5)
	University diploma	9 (7.9)	4 (7.0)	5 (8.8)
	University diploma or above	11 (9.6)	6 (10.5)	5 (8.8)
**Residential status**	Lives alone	14 (12.3)	6 (10.5)	8 (14.0)
	Lives with family	84 (73.7)	43 (75.4)	41 (71.9)
	Lives with friends	7 (6.1)	3 (5.3)	4 (7.0)
	Homeless	6 (5.3)	3 (5.3)	3 (5.3)
	Other	3 (2.6)	2 (3.5)	1 (1.8)
**Number of cohabitants**	≤ 3	67 (58.8)	36 63.2)	31 (54.4)
	4–6	38 (33.3)	17 (29.8)	21 (36.8)
	7–9	7 (6.1)	2 (3.5)	5 (8.8)
	≥ 10	2 (1.8)	2 (3.5)	0 (0)
**Bedrooms**	1	72 (63.2)	37 (64.9)	35 (61.4)
	2	30 (26.3)	14 (24.6)	16 (28.1)
	3	7 (6.1)	4 (7.0)	3 (5.3)
	≥ 4	5 (4.4)	2 (3.5)	3 (5.3)
**Household head**	Yes	63 (55.3)	33 (57.9)	30 (52.6)
	No	51 (44.7)	24 (42.1)	27 (47.4)
**Residency status**	Permanent	92 (80.7)	43 (75.4)	49 (85.9)
	Temporary	22 (19.3)	14 (24.6)	8 (14.0)
**Smoking per day**	Never	94 (82.5)	50 (87.7)	44 (77.2)
	1–5	19 (16.7)	6 (10.5)	13 (22.8)
	6–10	1 (0.9)	1 (1.8)	0 (0)
**Khat (a stimulant)**	Never	91 (79.8)	47 (82.5)	44 (77.2)
	1/week	10 (8.8)	3 (5.3)	7 (12.3)
	> = 2/week	9 (7.9)	5 (8.8)	4 (7.0)
	1/month	4 (3.5)	2 (3.5)	2 (3.6)
**Alcohol**	Never	72 (63.2)	40 (70.2)	32 (56.1)
	> 1/day	12 (10.5)	5 (8.8)	7 (12.3)
	2–5/day	19 (16.7)	7 (12.3)	12 (21.1)
	≥ 6/day	3 (2.6)	1 (1.8)	2 (3.5)
	Rarely	8 (7.0)	4 (7.0)	4 (7.0)
**HIV**^a^	Negative	96 (85.0)	47 (82.5)	49 (87.5)
	Positive	17 (15.0)	10 (17.5)	7 (12.5)
**On antiretroviral (If HIV positive)**	Yes	12 (73.3)	7 (70.0)	5 (71.4)
	No	5 (26.7)	3 (30.0)	2 (28.6)
**TB treatment**	New	102 (89.5)	50 (87.7)	52 (91.2)
	Relapse	12 (10.5)	7 (12.3)	5 (8.8)
**Place of diagnosis**	Study facility	78 (68.4)	36 (63.2)	42 (73.7)
	Health center	4 (3.5)	2 (3.5)	2 (3.5)
	Public hospital	10 (8.8)	8 (14.0)	2 (3.5)
	Private clinic/hospital	21 (18.4)	10 (17.5)	11 (19.3)
	Other	1 (0.9)	1 (1.8)	0 (0)
**TB test methodology**	Microscopy	44 (38.6)	18 (31.6)	26 (45.6)
	Xpert MTB/RIF	70 (61.4)	39 (68.4)	31 (54.4)
**Microscopy result (if test with microscopy)**^b^	1–9 (scanty)	1 (2.4)	0 (0)	1 (4.0)
	1+	10 (23.8)	5 (29.4)	5 (20.0)
	2+	15 (35.7)	10 (58.8)	5 (20.0)
	3+	16 (38.1)	2 (11.8)	14 (56.0)
**Completed treatment (if ever treated for TB)**	Yes	12 (100)	7 (100)	5 (100)

*TB* tuberculosis, *ARV* antiretroviral, *MTB/RIF* Mycobacterium tuberculosis/resistance to rifampicin, ^a^1 missing value; ^b^2 missing values

### Adherence to TB medications

#### Adherence primary analysis

Of 57 participants in the control (DOT) arm, 30 (52.6%) requested to take home some doses, of which the providers allowed this procedure for extenuating circumstances and provided doses for 29 (97%) participants on at least one occasion. These periods of take-home doses ranged from 3 to 15 days. There were 77 requests from the 29 participants on different occasions, with a minimum of one and a maximum of eight requests per participant. Among these, the providers agreed and provided doses for 74 (96%) of the requests. Of the total 3420 tablets expected to be administered in-person within the healthcare facilities, 1004 (29.4%) were taken home for self-administration.

For the primary endpoint that assumed all take-home doses in the control arm were ingested, the GM percentage adherence to anti-TB medication doses was 98.97% (GSD 1.04) in the control arm and 99.01% (GSD 1.02) in the intervention arm, with a non-significant difference between the two arms (mean ratio [MR] 1.00 [95% CI 0.99-1.01]; *p* = *0*.*954*) (Table 2).

**Table 2:** Comparison of treatment adherence between intervention and control arms (*n* = 114)

Arm	Adherence GM %	GSD	MR (95%CI)	***P value***
Intervention (*n* = 57)	99.01	1.02	1.00 (0.99-1.01)	*0.954*
Control (*n* = 57)	98.97	1.04	1	

*GM* geometric mean, *GSD* geometric standard deviation, *MR* mean ratio, *adherence* assumed all take-home doses ingested

**Fig. 3:**
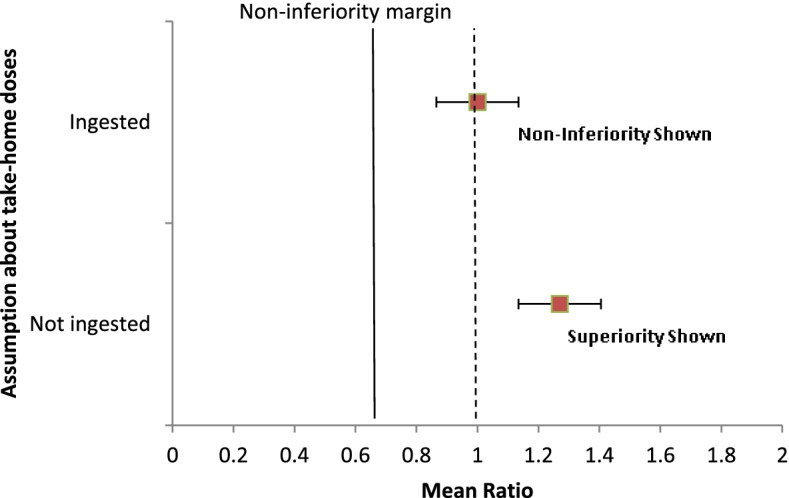
Dot plot of the mean ratio between intervention vs. control (all take-home doses ingested or no take-home doses ingested) arms (*n* = 114)

With this analysis, the intervention did not show superiority over the DOT control when considering all take-home doses were ingested, while the intervention still demonstrated that it was non-inferior to DOT. Figure 3 demonstrates these findings in a dot plot.

Using a Fisher’s exact test, the two arms had no significant difference in the proportion of participants who achieved an adherence threshold of ≥ 90% (*p* = *0.496*), and all participants had achieved an adherence threshold ≥ 80% (Table 3).

**Table 3:** Comparison of the proportion of participants who achieved adherence threshold ≥ 80% and ≥ 90% between intervention and control arms on Fisher’s exact test (*n* = 114)

Adherence level	Categories	Study arm		***P value***
		Intervention, *f* (%)	Control, *f* (%)	
**≥ 90%**	Achieved	57 (100)	55 (96.5)^a^	*0.496*
	Not achieved	0 (0)	2 (3.5)	
**≥ 80%**	Achieved	57 (100)	57 (100)	*NA*
	Not achieved	0 (0)	0 (0)	

^a^Assumed all take-home doses were ingested

A general linear model regression was used to determine the effect of sociodemographic, behavioral, and health characteristics of participants on their levels of adherence, with adjustments made by controlling other covariates. Results of the multivariable regression model revealed that HIV co-infection, at least one negative urine isoniazid test, and occupation were associated with level of adherence and the level of adherence was higher among HIV-negative compared with HIV-positive participants. The level of adherence was higher among participants who were employed compared with those unemployed. The level of adherence was higher among participants who had no negative urine isoniazid test compared with those with at least one negative result (Table 4). The GM percentage adherence as prescribed (standard DOT) or take-home in the control arm was 98.97 (GSD 1.04), having no significant difference with the intervention arm (AMR 0.99 [95% CI 0.98–1.00]; *p* = 0.189).

**Table 4:** Mean ratio and adjusted mean ratio for treatment adherence according to sociodemographic characteristics (*n* = 113)

Variables	Categories (***n***)	GM (GSD)	CMR (95%CI)	***P value***	AMR (95%CI)	***P value***
**Arm**	Intervention (57)	99.01 (1.02)	1.00 (0.99–1.01)	*0.928*	0.99 (0.98–1.00)	*0.189*
	Control (56)	98.97 (1.04)	1		1	
**Sociodemographic characteristics**						
Gender	Female (40)	99.11 (1.02)	1.00 (0.99–1.01)	*0.749*		
	Male (73)	98.92 (1.03)	1			
Age	-		0.99 (0.99–1.00)	*0.136*	0.99 (0.99–1.00)	*0.218*
Marital status	Married (51)	99.40 (1.02)	1.01 (0.99–1.02)	*0.175*	1.01 (0.99–1.02)	*0.116*
	Unmarried (62)	98.65 (1.04)	1		1	
Occupation	No job (36)	97.93 (1.05)	0.98 (0.97–0.99)	*0.006*	0.99 (0.98–0.99)	*0.023*
	Have job (77)	99.49 (1.02)	1		1	
Education	Below prep (82)	98.74 (1.03)	0.99 (0.98–1.00)	*0.160*	0.99 (0.98–1.00)	*0.214*
	Prep and above (31)	99.61 (1.01)	1		1	
# of people	≤3 (66)	99.13 (1.02)	1.00 (0.99–1.01)	*0.509*		
	≥4 (47)	98.76 (1.04)	1			
# of bedrooms	1 (71)	99.03 (1.02)	1.00 (0.99–1.01)	*0.823*		
	≥2 (42)	98.90 (1.04)	1			
Household head	No (50)	98.90 (1.03)	0.99 (0.98–1.01)	*0.788*		
	Yes (63)	99.06 (1.03)	1			
Residency	Permanent (91)	98.99 (1.03)	0.99 (0.98–1.01)	*0.995*		
	Temporary (22)	98.99 (1.02)	1			
**Behavioral characteristics**						
Smoking per day	Never (94)	99.06 (1.03)	1.00 (0.99–1.02)	*0.565*		
	Yes (19)	98.63 (1.03)	1			
Khat	Never (91)	98.90 (1.03)	0.99 (0.98–1.01)	*0.578*		
	Yes (22)	99.29 (1.02)	1			
Alcohol	Never (72)	99.15 (1.02)	1.00 (0.99–1.02)	*0.442*		
	Yes (41)	98.69 (1.04)	1			
**Disease conditions**						
HIV	Negative (96)	99.31 (1.02)	1.02 (1.01–1.04)	*0.005*	1.02 (1.01–1.04)	*0.003*
	Positive (17)	97.21 (1.06)	1		1	
TB treatment	New (102)	98.97 (1.03)	0.99 (0.98–1.02)	*0.778*		
	Relapse (11)	99.22 (1.02)	1			
At least one -ve urine isoniazid	No (100)	99.31 (1.02)	1.03 (1.01–1.05)	*<0.001*	1.03 (1.01–1.04)	*0.001*
	Yes (13)	96.47 (1.07)	1		1	

*CMR* crude mean ratio, *AMR* adjusted mean ratio, adjusted for all other covariates included in the model, *n* = 113 as 1 missing for HIV; *Prep* preparatory, *#* number, *-ve* negative

#### Adherence secondary analysis

A secondary analysis was conducted to determine the effect of take-home doses on the level of adherence for the DOT participants, which was compared with the intervention arm. The analysis had two assumptions: all take-home doses ingested or no take-home doses ingested. The 20% non-inferiority margin, which was equivalent to 12 pill count, was transformed to its equivalent ratio of 0.79 using e^−0.23^ as the data were log-transformed in the case of the count model. The assumed average adherence was 79% (47.4 pill count) vs 99% (59.4 pill count) within the given range of 20% non-inferiority. Assuming all take-home doses were not ingested, the GM percentage adherence as prescribed (standard DOT) was 77.71 (GSD 1.57), having a significant difference with the intervention arm (MR 1.27 [95% CI 1.33–1.43]; *p < 0*.*001*). With this analysis, the intervention showed superiority over the DOT control when considering all take-home doses were not ingested. The intervention demonstrated that it was non-inferior to DOT with both assumptions (all take-home doses ingested or not). In detail, (1) assuming no take-home doses were ingested, the intervention was superior to the DOT control, and (2) assuming all take-home doses were ingested, the intervention was not superior but was non-inferior to the DOT control (Fig. 3).

#### Adherence sensitivity analysis based on pill count

A sensitivity analysis was done considering pill count (number of pills taken during the two months intensive period) as the outcome, assuming all take-home doses with the control arm were ingested. Poisson and negative binomial regression were done to identify factors that determine participants’ level of adherence. The Poisson regression was selected as it has the smallest Akaike information criterion (AIC = 678.911) compared with the negative binomial regression (AIC = 680.911) and the dispersion parameter in negative binomial regression was not significantly different from zero. None of the variables were found to be potential candidates for the multiple regression. The regression revealed that the intervention was non-inferior to the control arm with rate ratio (RR) of 1.00 (95% CI: 0.95–1.05), given a non-inferiority margin of 0.82 (= e^-0.2^) (Table 5).

**Table 5:** Poisson regression analysis of crude rate ratio for treatment adherence according to sociodemographic characteristics on (*n* = 113)

Variables	Categories (***n***)	Mean (SD)	CRR (95%CI)	***P value***
**Arm**	Intervention (57)	59.44 (1.29)	1.00 (0.95–1.05)	*0.985*
	Control (56)	59.41 (1.97)	1	
**Sociodemographic characteristics**				
Gender	Female (40)	59.48 (1.19)	1.00 (0.95–1.05)	*0.959*
	Male (73)	59.39 (1.87)	1	
Age	-		1.00 (0.99–1.00)	*0.773*
Marital status	Married (51)	59.65 (1.02)	1.01 (0.96–1.06)	*0.781*
	Unmarried (62)	59.24 (2.02)	1	
Occupation	No job (36)	58.81 (2.49)	0.99 (0.94–1.04)	*0.559*
	Have job (77)	59.71 (0.96)	1	
Education	Below prep (82)	59.29 (1.88)	0.99 (0.94–1.05)	*0.992*
	Prep and above (31)	59.77 (0.76)	1	
# of people	≤ 3 (66)	59.52 (1.35)	1.00 (0.96–1.05)	*0.883*
	≥ 4 (47)	59.29 (2.02)	1	
# of bedrooms	1 (71)	59.45 (1.38)	1.00 (0.95–1.05)	*0.963*
	≥ 2 (42)	59.38 (2.06)	1	
Household head	No (50)	59.36 (1.51)	0.99 (0.95–1.05)	*0.937*
	Yes (63)	59.48 (1.78)	1	
Residency	Permanent (91)	59.43 (1.72)	1.00 (0.94–1.06)	*0.992*
	Temporary (22)	59.41 (1.40)	1	
**Behavioral characteristics**				
Smoking per day	Never (94)	59.48 (1.62)	1.00 (0.94–1.07)	*0.920*
	Yes (19)	59.26 (1.88)	1	
Khat	Never (91)	59.37 (1.77)	0.99 (0.94–1.06)	*0.886*
	Yes (22)	59.63 (1.05)	1	
Alcohol	Never (72)	59.51 (1.19)	1.00 (0.96–1.06)	*0.871*
	Yes (41)	59.27 (2.27)	1	
**Disease conditions**				
HIV	Negative (96)	59.59 (1.19)	1.02 (0.95–1.09)	*0.580*
	Positive (17)	58.47 (3.10)	1	
TB treatment	New (102)	59.40 (1.72)	0.99 (0.92–1.08)	*0.924*
	Relapse (11)	59.63 (0.92)	1	
At least one -ve urine isoniazid	No (100)	59.61 (1.06)	1.03 (0.95–1.11)	*0.479*
	Yes (13)	58.00 (3.72)	1	

*CRR* crude rate ratio, *n* = 113 as 1 missing for HIV, *Prep* preparatory, *#* number, *-ve* negative

A sensitivity analysis was done considering pill count as the outcome, assuming all take-home doses with the control arm were not ingested. Poisson and negative binomial regressions were done to identify factors determining participant level of adherence. The negative binomial regression was selected as it has the smallest AIC (903.114) compared with the Poisson regression (AIC = 933.443), and the dispersion parameter in negative binomial regression was significantly different from zero. The multiple regression revealed that study arm, occupation, and at least one negative urine isoniazid test result were significant factors determining participants’ level of adherence. Given the non-inferiority margin of 0.82 (= e^−0.2^), the intervention demonstrates superiority as well as non-inferiority (Table 6).

**Table 6:** Negative binomial regression analysis of crude rate ratio and adjusted rate ratio for treatment adherence according to sociodemographic characteristics (*n* = 113)

Variables	Categories (***n***)	Mean (SD)	CRR (95%CI)	***P value***	ARR (95%CI)	***P value***
**Arm**	Intervention (57)	59.44 (1.29)	1.19 (1.09–1.29)	*<0.001*	1.16 (1.07–1.26)	*<0.001*
	Control (56)	49.98 (14.68)	1		1	
**Sociodemographic characteristics**						
Gender	Female (40)	53.15 (14.21)	0.96 (0.89–1.02)	*0.162*	1.02 (0.95–1.09)	*0.658*
	Male (73)	55.63 (9.45)	1		1	
Age	-		0.99 (0.99–1.00)	*0.140*	0.99 (0.99–1.00)	*0.704*
Marital status	Married (51)	55.88 (10.29)	1.04 (0.98–1.10)	*0.229*		
	Unmarried (62)	53.82 (12.18)	1			
Occupation	No Job (36)	50.00 (15.99)	0.88 (0.79–0.97)	*0.007*	0.89 (0.82–0.98)	*0.012*
	Have job (77)	56.97 (7.54)	1		1	
Education	Below Prep (82)	53.77 (12.82)	0.94 (0.88–1.00)	*0.061*	0.93 (0.86–1.02)	*0.108*
	Prep and above (31)	57.35 (5.38)	1		1	
# of people	≤ 3 (66)	56.00 (10.95)	1.06 (0.99–1.12)	*0.082*	1.04 (0.98–1.11)	*0.225*
	≥ 4 (47)	53.00 (11.82)	1		1	
# of bedrooms	1 (71)	54.72 (10.95)	0.99 (0.94–1.06)	*0.959*		
	≥ 2 (42)	54.81 (12.17)	1			
Household head	No (50)	53.76 (12.09)	0.97 (0.91–1.03)	*0.299*		
	Yes (63)	55.54 (10.79)	1			
Residency	Permanent (91)	54.37 (11.52)	0.97 (0.89–1.04)	*0.368*		
	Temporary (22)	56.31 (10.81)	1			
**Behavioral characteristics**						
Smoking per day	Never (94)	55.05 (10.84)	1.03 (0.95–1.12)	*0.430*		
	Yes (19)	53.26 (13.91)	1			
Khat	Never (91)	54.65 (11.83)	0.99 (0.92–1.07)	*0.804*		
	Yes (22)	55.18 (9.42)	1			
Alcohol	Never (72)	55.09 (11.19)	1.02 (0.96–1.08)	*0.591*		
	Yes (41)	54.15 (11.78)	1			
**Disease conditions**						
HIV	Negative (96)	54.39 (11.82)	0.96 (0.88–1.04)	*0.322*		
	Positive (17)	56.76 (8.36)	1			
TB treatment	New (102)	54.35 (11.86)	0.93 (0.84–1.03)	*0.157*	0.97 (0.87–1.07)	*0.502*
	Relapse (11)	58.45 (3.05)	1		1	
At least one -ve urine isoniazid	No (100)	56.01 (10.19)	1.24 (1.12–1.37)	*< 0.001*	1.15 (1.00–1.31)	*0.043*
	Yes (13)	45.08 (15.31)	1		1	

*CRR* crude rate ratio, *ARR* adjusted rate ratio, *n* = 113 as 1 missing for HIV, *Prep* preparatory, *#* number, *-ve* negative

### IsoScreen^TM^ urine Isoniazid test

Urine isoniazid colorimetric assay was performed on 443 (97%) samples out of the expected 456, of which 430 (97%) tested positive, indicating that the drug was taken within the 24–30 h prior to the urine test. There were 14 negative results, of which 12 were from control and two from intervention arms. The urine test was performed four times for 106 (93%) of the 114 participants, while the remaining eight participants missed a total of 13 samples: two participants from the control arm missed three samples each; one participant from the intervention arm missed two samples; and five participants, one from control and four from intervention arms, missed one sample each.

Thirteen (11.4%) participants had at least one negative result [control 11 (19.3%) vs intervention 2 (3.5%)]. Log binomial regression was done to estimate the risk of study arms for a negative ionized urine test. There was a significant association between the urine isoniazid test and study arms (*p* = *0.022*). Participants in the DOT arm were more likely to have a negative isoniazid urine test, and the risk of having at least one negative urine isoniazid test in the DOT arm was 5.50 times more likely compared with the intervention arm (Table 7).

**Table 7:** Comparison of urine isoniazid test results between intervention and control arms (*n* = 114)

Variable	Categories	4x urine isoniazid test result		RR (95% CI)	***p-value***
		≥1 neg *f* (%)	4x pos *f* (%)		
**Treatment arm**	Intervention	2 (3.5)	55 (96.5)	1	*0.022*
	Control	11 (19.3)	46 (80.7)	5.50 (1.28–23.71)	
	Total	13 (11.4)	101 (88.6)		

*RR* risk ratio, *neg* negative, *pos* positive, *f* frequency

**Fig. 4:**
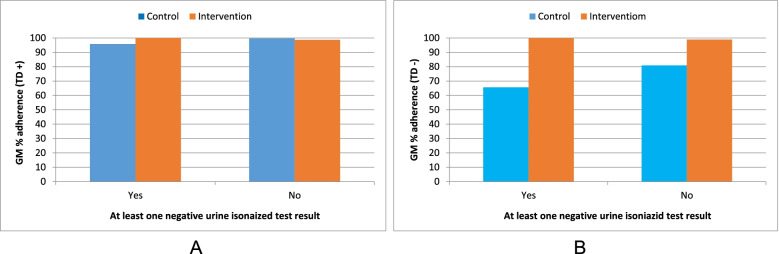
Geometric mean % adherence by urine isoniazid test results between the intervention vs. control (assuming all take-home doses ingested [TD+, A] or no take-home doses ingested [TD−, B]) arms (*n* = 114)

In the control arm, the mean adherence was higher for those who had no negative urine test results compared with those who have at least one negative result. In the intervention arm, the average adherence was the same regardless of the urine test results and was lower than in the control arm (Fig. 4).

### Adherence self-report

Adherence self-report data were captured at the end of the intensive phase for 109 (95.6%) of the total 114 participants to identify the relationship between self-report and objective adherence measures over this 2-month period. Six (5.6%) reported they often forgot to take their TB medications; the difference between the two arms did not reach statistical significance [control 5 (8.9%) vs intervention 1 (1.9%), *p* = *0*.*21*]. The adherence self-report had a significant association with the actual adherence results (*p* = *0*.*013*). Twenty (18.5%) participants felt stressed about meeting their TB treatment schedule, with no significant difference between the two arms [control 12 (21.4%) vs intervention 8 (15.4%), *p* = *0*.*42*]. The adherence self-report had a significant association with the actual adherence result (*p* = *0*.*013*) but not with the urine isoniazid test (*p>0.99*).

### Sputum smear conversion

Smear conversion data were captured at the end of the intensive phase. Microscopy was performed for 108 (94.7%) participants, of whom 107 (99.1%) were negative with smear microscopy and were transitioned into the four-month continuation phase, while one (1%) remained positive and was treated for Xpert MTB/RIF-proven drug-resistant-TB per the national guidelines. There was no statistically significant difference between the arms on smear negativity following the intensive phase [control 55 (98.2%) vs intervention 52 (100%), *p* = *0.99*].

### Adverse treatment outcomes

Adverse treatment outcomes data were captured from enrolment. One participant from the intervention arm was lost-to-follow-up. The participant (female, 18 years old, commercial sex worker, HIV negative, new TB case) was lost to follow-up after completing the first month of treatment and taking 15 days of doses for the second month. Her two urine isoniazid tests were positive, and based on the data downloaded from the pillbox device, she had taken her first month of treatment as prescribed. There was no statistically significant difference between the arms for the adverse treatment outcome measures including death [control 0 vs intervention 1 (1.9%), *p* = *0*.*48*].

## Discussion

In this randomized controlled trial of patients with drug-susceptible pulmonary TB, MERM-enabled self-administered therapy showed non-inferior treatment adherence compared with the standard in-person DOT. This suggests that the use of electronic MERM, which holds TB medication supply, reminds patients using audible and visual alarms to self-administer their daily medication and refill medications and records medication intake events is a good alternative to in-person DOT. There was a significant association between the urine isoniazid test results and treatment adherence. Patients in the intervention arm were less likely to have a negative isoniazid urine test. The risk of having at least one negative urine isoniazid test was more likely for participants receiving in-person DOT compared with MERM-enabled self-administered therapy. There was a significant association between overall self-report adherence and actual adherence; however, there was no statistically significant difference between the two arms for self-report adherence. There was no statistically significant difference between the two arms for smear negativity following the intensive phase or on adverse treatment outcomes including lost-to-follow-up and death. This was likely due to the routinely practiced take-home of doses for participants in the control arm when the provider approved these exceptions for extenuating circumstances. Based on their experience, providers believed that denying these requests and insisting on strict DOT would lead to missed doses and thus result in lower rates of smear conversion and successful treatment.

To our knowledge, this trial is the first study to apply a randomized trial design to investigate the effectiveness of a MERM-observed self-administered therapy for patients with TB in an African setting and the second such study globally. Only one other pragmatic cluster-randomized trial has been published and reported a significantly improved medication adherence with the use of MERM in China [10], which was consistent with our findings in the secondary endpoints, but not the primary endpoint. The randomized controlled trial in the USA found a non-inferior adherence to TB medications with the use of wireless DOT [11] which was in agreement with our findings in the primary endpoint, but not the secondary endpoints. The pragmatic stepped-wedge cluster-randomized trial on 99DOTS-based treatment monitoring in Uganda [9] and the retrospective cohort study on electronic medication monitor in China [13] did not show improvement in TB treatment outcomes with the use of digital health technologies.

In this trial, treatment adherence in the intervention arm was non-inferior compared with the control arm, suggesting that it is possible to monitor participants during their treatment but without the need for daily visits to a healthcare facility. Daily facility visits are conducted during the 2-month intensive phase where their early disease condition, travels, and economic status could be barriers and interrupt treatment. Compared with the intensive phase, the 4-month continuation phase may present less of a burden as visits are weekly. Forgetfulness, lack of support, fear of drug side-effects, and lack of hope were major reasons for non-adherence. The findings revealed a significant association between urine isoniazid test and treatment adherence, unlike a previous prospective cohort study in India that reported a suboptimal accuracy of 99DOTS-based electronic medication monitor compared with urine isoniazid partly due to poor patient engagement with 99DOTS [12].

This trial revealed a suboptimal implementation of in-person DOT, which concurs with previous studies conducted in Ethiopia [22, 26]. We explored whether in-person DOT participants might take-home some doses. A significant number of participants needed to take-home some doses for personal reasons and providers permitted this, while there was no assurance that such doses were actually administered. These participants completed their intensive phase and experienced smear conversion. However, there was a significant difference in urine isoniazid test positivity between the intervention and the control participants, implying that a participant in the DOT arm could take-home some doses to self-administer during challenging periods but actual administration could not be ensured. Although our study findings show that MERM-observed self-administered therapy resulted in non-inferior adherence, and superior by some measures when compare with in-person DOT, further research is needed to understand whether adherence in the intervention was due to allowing self-administration of therapy, which greatly reduced the burden of clinic visits, the support provided by the MERM system, or both. Future randomized trials comparing MERM-observed self-administered therapy to self-administered therapy alone may help determine whether the MERM system provides additional value for promoting adherence.

Adherence self-report data were captured at the end of the intensive phase for 109 participants, and the analysis showed a significant association between adherence self-report and the actual adherence result. This implies that self-report can be a potential option to measure medication adherence in this setting. Self-report adherence was high in both arms, with only six (5.6%) participants from both arms reported they often forget to take their TB medication, and with no significant difference between the two arms. Moreover, 20 (18.5%) participants overall felt stressed about meeting their TB treatment schedule, but there was no significant difference between groups.

This trial also demonstrated that TB is predominantly a disease of the disadvantaged and that comprehensive economic and social protection is required to ensure successful treatment adherence and completion. The participants’ mean monthly income was ETB 2959.21, which was lower than the national average of ETB 8074 [33]. Additionally, 63.2% live in a house with a single bedroom, and 5.3% were homeless. The percentage of participants co-infected with HIV (15%) was higher than the national and global prevalence of 8%. The analysis showed that having a job has a beneficial effect on the level of adherence. There was no association between substance use behaviors and the level of medication adherence or treatment outcomes. The level of adherence was lower for participants with HIV compared with participants without HIV. In the trial, 10.5% of participants were relapsed, and there was no significant association between treatment adherence and whether participants had a prior relapse.

The vast majority of TB research studies were at least partially affected by the COVID-19 pandemic. In Ethiopia, the COVID-19 pandemic significantly interrupted routine TB care and treatment services and reduced the rate of TB case-detection. Several articles have been published informing local governments of interventions to retain patients with TB in care [34], the implications of COVID-19 on the SELFTB trial along with strategies employed to mitigate these barriers [35], and the real-time impact of COVID-19 on clinical care and treatment of patients with TB across the various trial sites [36].

Our study has several limitations. The overall sample size was modest. The approaches followed to measure the primary outcome differed according to the study arm (MERM data versus DOT records) that may lead to ascertainment bias. However, this is the conventional approach used for comparing medication event monitors data to standard of care in clinical trial settings. This study was carried out in Addis Ababa, Ethiopia, and this limits the generalizability of our results. Adherence is a multidimensional phenomenon determined by multiple sets of factors that may have biased our findings. Patients are given a fixed-dose combination of anti-TB medication where the findings may not apply in locations that do not use this. We did not use pre-existing scales to measure adherence self-report which may hinder the validity of the measurement. Blinding was not feasible. End-of-treatment outcomes are necessary to demonstrate the impact of DAT among patients with TB more broadly. The use of an individual participant randomized trial, instead of a pragmatic trial, may limit the findings to inform decision-making. However, despite these limitations, we trust the study reveals important findings. We used multiple measurement tools to assess medication adherence, which strengthened the findings. Furthermore, we conducted the study in Ethiopia, one of the countries with the highest burden of TB but poorly represented in such clinical trials. In our recent systematic review of the literature [27], we found that DATs hold much promise in strengthening healthcare systems in Ethiopia, while the use of such technologies was a relatively new phenomenon and randomized trials were critically limited.

## Conclusions

In this randomized trial of patients with drug-sensitive pulmonary TB, medication adherence among participants assigned to MERM-observed self-administered therapy was non-inferior and superior by some measures when compared with standard in-person DOT. Further research is needed to understand whether adherence in the intervention is primarily driven by allowing self-administered therapy which reduced challenges of repeated clinic visits or by the adherence support provided by the MERM system. To avoid contributing to patient barriers with DOT, TB medical programs should consider alternatives such as medication event monitors.

## Supplementary Information

**Additional file 1.** Study protocol.

**Additional file 2.** CONSORT checklist.

**Additional file 3.** Baseline questionnaire for TB patients.

**Additional file 4.** Patients user leaflet of evriMED500.

**Additional file 5.** Treatment adherence monitoring tool.

## Data Availability

The data that support the findings of this study are available upon reasonable request from the corresponding author.

## Abbreviations

DAT: Digital adherence technologies
DOT: Directly observed therapy
2HRZE: 2RHZE (rifampicin [R]/150 mg + isoniazid [H]/75 mg + pyrazinamide [Z/400 mg + ethambutol ([E]/275 mg)
ITT: Intention-to-treat
MERM: Medication event reminder monitor system
TB: Tuberculosis
WHO: World Health Organization
