# Supplementary material for: Effectiveness of a digital medication event reminder and monitor device for patients with tuberculosis (SELFTB): a multicenter randomized controlled trial
Source: BMC Med. 2022 Sep 28;20:310. doi: 10.1186/s12916-022-02521-y (PMC9514884; doi:10.1186/s12916-022-02521-y)
Supplement: Supplementary file 3 — Additional file 3. Baseline questionnaire for TB patients. [file 12916_2022_2521_MOESM3_ESM.pdf]

Baseline questionnaire for TB patients

Participant ID: ☐☐☐☐☐☐ Arm ☐SAT ☐DOT Health Professional ID: ☐☐☐☐			
Demographics			
Sex: ☐Male ☐Female<br>Age in years: ☐☐<br>Region …………………..<br>Zone/Sub-city ………..	Occupation:<br>☐No job ☐Housewife<br>☐Student ☐Government<br>☐Farmer ☐Daily laborer<br>☐Trader ☐Other …….	Educational Level:<br>☐None<br>☐Secondary (9-10)<br>☐University (Diploma)	☐Elementary (0-8)<br>☐Preparatory (11-12)<br>☐University (≥Degree)
Marital Status:<br>☐Married ☐Never<br>☐Divorced<br>☐Widower	Whom you live with?<br>☐Alone ☐Family<br>☐Friends ☐Homeless<br>☐Other ……	How many people live in the house including you?<br>☐≤ 3 ☐4-6<br>☐7-9 ☐≥ 10	How many bedrooms are in the house?<br>☐1 ☐2<br>☐3 ☐≥4
Are you head of the household?<br>☐Yes ☐No	Are you a permanent or temporary resident in Addis<br>☐Permanent ☐Tempo.	What is your average monthly income?<br>………………………..	Distance from home to this health center<br>………………….. KM
Behavioral			
Smoking cigarettes per day<br>☐Never ☐1-5<br>☐ 6-10 ☐>10	Use of Chat:<br>☐Never ☐1/week<br>☐≥2/week ☐1/month	Drinking alcohol:<br>☐Never ☐<br>>1/day;<br>☐2-5/day ☐ ≥6/day<br>☐Weekly ☐Rarely	Taking cocaine/marijuana:<br>☐Never<br>☐Occasionally<br>☐ Regularly, <5/day<br>☐ Regularly, >5/day
From TB registration log			
HIV status:<br>☐Pos ☐Neg	If HIV+, currently on ARV<br>☐Yes ☐No	Weight in Kg …………<br>Height in cm …………	Date TB treatment started (DD-MM-YY)<br>……………………………….
TB treatment status<br>☐new ☐relapse<br>Date diagnosed as TB patient …………………….	TB tested positive using:<br>☐Microscopy<br>☐Xpert MTB/RIF<br>If microscopy, result was:<br>☐1-9(Scanty) ☐1+<br>☐2+ ☐3+	If drug susceptibility test done, result<br>☐Susceptible<br>☐Resistant<br>Have you ever treated	Place of diagnosis:<br>☐This facility<br>☐Another health center<br>☐Public hospital<br>☐NGO health facility<br>☐Private clinic/ hospital<br>☐Other………………
Have you ever had TB treatment before?<br>☐Yes ☐No	If yes: Have you completed your previous TB treatment?<br>☐Yes ☐No, why ……..		
**Study clinician signature**		**Date (Ethiopian calendar):**	
