# Supplementary material for: Effectiveness of a digital medication event reminder and monitor device for patients with tuberculosis (SELFTB): a multicenter randomized controlled trial
Source: BMC Med. 2022 Sep 28;20:310. doi: 10.1186/s12916-022-02521-y (PMC9514884; doi:10.1186/s12916-022-02521-y)
Supplement: Supplementary file 4 — Additional file 4. Patients user leaflet of evriMED500. [file 12916_2022_2521_MOESM4_ESM.pdf]

መመሪያዎች

1. ያጠቃቀም መመሪያዎች

1. መድሃኒት የሚወሰዱበትን ጊዜ ለማስታወስ ሳጥኑ ከድምጽ ጋር በመቀናጀት አረንጋዴ መብራት ያበራል።

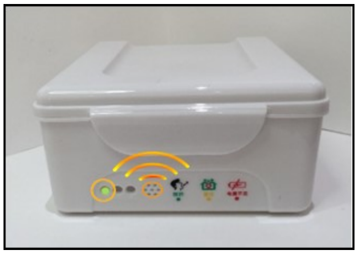

2. መድኃኒቶን ለመዋጥ መዝጊያ ክዳኑን በማንሳት ሳጥኑን ይክፈቱ።

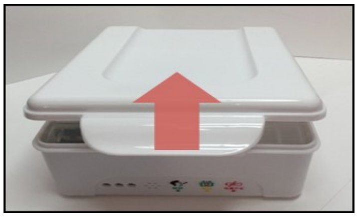

3. ከሐኪም በተነገረው መሰረት መድሃኒትዎን ይዋጡ።

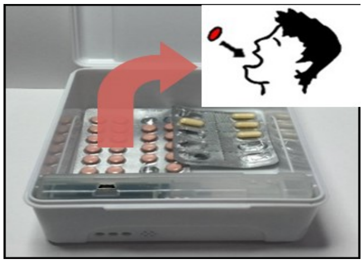

4. መድሃኒትዎን ወስደው ሲጨርሱ ሳጥኑን በመክደን ደህንነቱ በሚጠበቅበት ቦታ ያስቀምጡት።

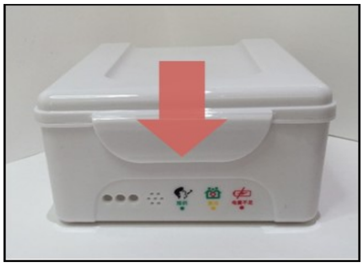

2. የሳጥኑ የመብራት ቀለሞች ምንነት

- አረንጓዴ መብራት እና ድምጽ ዕለታዊ መድሃኒት አስታዋሽ ምልክት ነው።

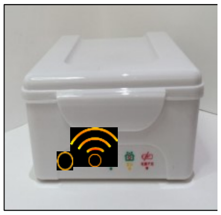

- ቢጫ መብራት መድሃኒትዎ ካለቀ ሕክምና ተቋም ሂደው እንዲሞሉ አስታዋሽ መብራት ነው።

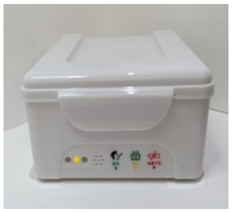

- ቀይ መብራት ዝቅተኛ የባትሪ ማንቂያ ማስጠንቀቂያ ነው።

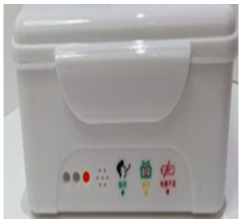

በየትኛውም ሁኔታ ያጋጠሞት ችግር ካለ ከቀጠሮ ቀን በፊትም ቢሆን በሳጥኑ ላይ ባለው ስልክ በመደወል የጤና ባለሞያ ያማክሩ።
