# Supplementary material for: Effectiveness of a digital medication event reminder and monitor device for patients with tuberculosis (SELFTB): a multicenter randomized controlled trial
Source: BMC Med. 2022 Sep 28;20:310. doi: 10.1186/s12916-022-02521-y (PMC9514884; doi:10.1186/s12916-022-02521-y)
Supplement: Supplementary file 5 — Additional file 5. Treatment adherence monitoring tool. [file 12916_2022_2521_MOESM5_ESM.pdf]

Treatment adherence monitoring tool

Adherence monitoring tool (MERM and DOT arms)

Participant ID: ☐☐☐☐☐☐ Arm ☐MERM ☐DOT Health Professional ID: ☐☐☐☐

Adherence follow-up

Month ☐1 ☐2

The patient visited the facility (DOT)/opened the box (MERM) and has taken the medication.
Check daily record (DOT)/every 15 days (MERM) and mark √ if taken, X if not taken

1	2	3	4	5	6	7	8	9	10	11	12	13	14	15	16	17	18	19	20	21	22	23	24	25	26	27	28	29	30

Reason for non-adherence, if any

Week 1: Any non-adherence? ☐ Yes ☐No

If yes, date/s ……………… # of days missed: ………..
Reason:

☐Forgetfulness 0 ☐Feel the dose given is high4
☐Drug side-effect1 ☐Finance for food/transport5
☐Ran out of pills2 ☐Lack of hope6
☐Lack of supporter3 ☐Feel drug is not effective7

Week 2: Any non-adherence? ☐ Yes ☐No

If yes, date/s …………………… # of days missed: ………..
Reason:

☐Forgetfulness ☐Feel the dose given is high
☐Drug side-effect ☐Finance for food/transport
☐Ran out of pills ☐Lack of hope
☐Lack of supporter ☐Feel drug is not effective

Week 3: Any non-adherence? ☐ Yes ☐No

If yes, date/s ………………… # of days missed: ………..
Reason:

☐Forgetfulness ☐Feel the dose given is high
☐Drug side-effect ☐Finance for food/transport
☐Ran out of pills ☐Lack of hope
☐Lack of supporter ☐Feel drug is not effective

Week 4: Any non-adherence? ☐ Yes ☐No

If yes, date/s …………………… # of days missed: ………..
Reason:

☐Forgetfulness ☐Feel the dose given is high
☐Drug side-effect ☐Finance for food/transport
☐Ran out of pills ☐Lack of hope
☐Lack of supporter ☐Feel drug is not effective

Urine pyrazinamide test results every 15 days

Urine test result	☐Positive ☐Negative
Urine test result	☐Positive ☐Negative

For DOT arm only
If a patient asks to take additional medication for the next day/s, fill:

Date asked: ……………………. For how many days asked: …………………. Measures taken: ……………………………
Date asked: ……………………. For how many days asked: …………………. Measures taken: ……………………………
Date asked: ……………………. For how many days asked: …………………. Measures taken: ……………………………
Date asked: ……………………. For how many days asked: …………………. Measures taken: ……………………………
